# Editorial: Unveiling inflammaging – mechanistic insights on aging and related diseases

**DOI:** 10.3389/fmed.2025.1607050

**Published:** 2025-04-22

**Authors:** Senthil Kumaran Satyanarayanan, Barani Kumar Rajendran

**Affiliations:** ^1^Centre for Regenerative Medicine and Health, Hong Kong Institute of Science & Innovation, Chinese Academy of Sciences, Hong Kong Science Park, Hong Kong, Hong Kong SAR, China; ^2^School of Medicine, Yale University, New Haven, CT, United States

**Keywords:** inflammaging, age-related diseases, immune dysregulation, therapeutic interventions, chronic inflammation

In the dynamic realm of gerontological research, a profound exploration into the intricate biological underpinnings of aging has unveiled groundbreaking insights. Among these, the biological phenomenon of “inflammaging,” referring to the chronic, low-grade inflammation accompanying aging, has emerged as a critical factor underlying age-associated pathologies. The current Research Topic “*Unveiling Inflammaging – Mechanistic Insights on Aging and Related Diseases*” encapsulates six diverse and mechanistically rich studies, each contributing to our understanding of inflammaging and its pathological consequences across systems. This editorial summarizes and synthesizes the principal findings of these contributions, highlighting how each study enhances our knowledge of inflammaging and its role in health span, disease susceptibility, and therapeutic opportunity. These articles are notably diverse, spanning from mechanistic reviews to original bioinformatics and epidemiological studies. They address aging across multiple physiological systems (neurological, renal, musculoskeletal, dermatological, and metabolic), illustrating the systemic reach of inflammaging. Moreover, the methodologies employed range from advanced data mining and machine learning to experimental and narrative reviews, providing a holistic overview of both basic science and translational avenues. This diversity is essential, as it reflects the complexity of inflammaging and underscores the need for interdisciplinary approaches to understanding and mitigating age-related diseases.

Liang et al. utilize a robust bioinformatics approach to identify and validate aging-related gene signatures in diabetic nephropathy (DN), a prototypical inflammaging-driven disorder. Through integration of GEO datasets and application of machine learning algorithms, the study identifies three diagnostic genes (EFEMP1, GHR, and VEGFA) that correlate with immune cell infiltration. The study shows a negative correlation between these aging markers and immune cell populations, particularly macrophages and T-cells, suggesting that inflammaging may disrupt immune surveillance and exacerbate renal pathology. Moreover, predicted microRNA and transcription factor interactions add a new layer to understanding the regulatory mechanisms involved in DN pathogenesis. This work provides a diagnostic framework for inflammaging in renal disease and opens avenues for precision medicine.

Yoshihara et al. shift the focus to periostin (POSTN), an ECM protein upregulated by mechanical stress and inflammation. Their review connects POSTN to the senescence-associated secretory phenotype (SASP) in spinal degenerative diseases (SDDs), such as intervertebral disc degeneration and ligament ossification. The authors convincingly argue that POSTN serves both as a mechanosensitive biomarker and as an active participant in tissue degeneration, linking mechanical stress, inflammation, and aging. POSTN's involvement in NF-κB, TGF-β, and PI3K/Akt signaling pathways aligns it closely with the inflammaging axis. Moreover, its expression in response to SASP factors positions it as a molecular bridge between aging cells and structural deterioration. The review elevates POSTN as a promising target for early diagnosis and therapeutic intervention in age-related musculoskeletal disorders.

Gou et al. employ data from NHANES to investigate the relationship between Life's Essential 8 (LE8), a composite cardiovascular health score, and the prevalence of metabolic syndrome (MetS) in older adults. The analysis reveals that higher LE8 scores are strongly protective against MetS, and biological aging, measured via serum Klotho levels and phenotypic age, partially mediates this relationship. Inflammatory indices such as the systemic immune-inflammation index (SII) and dietary inflammatory index (DII) are also implicated as intermediaries. This study elegantly links modifiable health behaviors to the molecular drivers of inflammaging, suggesting that lifestyle optimization may attenuate systemic inflammation and slow age-related metabolic decline.

Du et al. provides a narrative review on the biological mechanisms and clinical benefits of general and traditional Chinese exercises (e.g., Tai Chi, Qigong) in managing knee osteoarthritis (KOA). The review highlights how these interventions modulate inflammatory cytokines, improve cartilage metabolism, and reduce oxidative stress and cellular senescence. Importantly, exercise is shown to influence the SASP and downregulate key inflammatory pathways like NF-κB and MAPK, supporting its role in mitigating inflammaging. The dual focus on Western and Eastern exercise modalities provides an integrative framework for non-pharmacological management of KOA, a disease emblematic of musculoskeletal inflammaging.

Dorf and Maciejczyk delve into the mechanisms of skin aging, emphasizing how mitochondrial ROS, telomere attrition, and ECM remodeling converge to produce the senescent skin phenotype. The review catalogs cellular biomarkers such as SA-β-gal, p16INK4a, and DNA-SCARS, while also discussing non-invasive diagnostic approaches, including skin autofluorescence for AGEs. The skin, as a visible and accessible organ, serves as a sentinel of systemic aging and inflammaging. This work highlights the translational potential of skin biomarkers in evaluating biological age and monitoring therapeutic efficacy in anti-aging interventions.

Satyanarayanan et al. presents a timely and comprehensive review exploring the role of Toll-like receptors (TLRs) in post-COVID-19-associated neurodegenerative disorders. The authors describe how SARS-CoV-2, through its neurotropic properties and immune dysregulation, may trigger or exacerbate neurodegenerative conditions such as Alzheimer's and Parkinson's disease. TLRs, especially TLR3 and TLR7, are identified as key mediators of neuroinflammation, bridging viral recognition to chronic immune activation. Notably, the review underscores how dysregulated TLR signaling may persist after infection, contributing to the neuroinflammatory milieu that drives long-term sequelae. These insights advance the idea that inflammaging may be accelerated by viral insults, with TLRs as potential therapeutic targets for post-COVID neurological outcomes.

Collectively, these six articles underscore the multifactorial nature of inflammaging, bridging diverse organ systems and molecular pathways. TLR signaling, senescence biomarkers, metabolic regulators, and lifestyle factors emerge as unifying themes that connect aging with chronic inflammation and tissue degeneration. Importantly, each study introduces potential diagnostic or therapeutic strategies, from molecular biomarkers to lifestyle-based interventions. As our population ages, the imperative to understand and modulate inflammaging becomes ever more pressing. The synthesis of these investigations brings us closer to decoding aging not merely as an inevitable decline, but as a modifiable trajectory.

